# Correction: The development and validation of the Videogaming Motives Questionnaire (VMQ)

**DOI:** 10.1371/journal.pone.0280007

**Published:** 2022-12-30

**Authors:** Francisco J. López-Fernández, Laura Mezquita, Mark D. Griffiths, Generós Ortet, Manuel I. Ibáñez

The raw non-aggregate data underlying the results of this article [1] are missing from the supporting information. The authors have provided the data through a public data repository.

The previous Data Availability statement is therefore incorrect. The correct statement is: The data that support the findings of this study are openly available in Open Science Framework (OSF) at https://osf.io/st8jc/.

With this correction, all relevant data are now provided.
