# Supplementary material for: The development and validation of the Videogaming Motives Questionnaire (VMQ)
Source: PLoS One. 2020 Oct 23;15(10):e0240726. doi: 10.1371/journal.pone.0240726 (PMC7584249; doi:10.1371/journal.pone.0240726)
Supplement: S1 Table — (DOCX) [file pone.0240726.s001.docx]

**S1 Table. Study 1: Pattern matrix for oblimin eight-factor solution extracted from pilot Videogamimg Motives Questionnaire (VMQ)**

| **Juego a videojuegos porque:**  ***I play video games because:*** | Recreation | Social interaction | Coping-escape | Violent reward | Fantasy | Customization | Competition | Skill development |
| --- | --- | --- | --- | --- | --- | --- | --- | --- |
| Disfruto jugando  *I enjoy gaming* | .72 |  |  |  |  |  |  |  |
| Me entretienen  *Games entertain me* | .66 |  |  |  |  |  |  |  |
| Me lo paso bien  *I have fun* | .65 |  |  |  |  |  |  |  |
| Es divertido  *It is entertaining* | .59 |  |  |  |  |  |  |  |
| Me gustan mucho  *I like them much* | .57 |  |  |  |  |  |  |  |
| Me relaja  *It relaxes me* | .56 |  |  |  |  |  |  |  |
| Alivia mi estrés  *It helps me get rid of stress* | .47 |  | .33 |  |  |  |  |  |
| Hago nuevos amigos  *I make new friends* |  | .89 |  |  |  |  |  |  |
| Me permite conocer a otras persones  *It allows me to meet other people* |  | .85 |  |  |  |  |  |  |
| Mediante el juego estoy en contacto con mis amigos  *I keep in touch with my friends by gaming* |  | .82 |  |  |  |  |  |  |
| Disfruto jugando en grupo  *I enjoy playing in group* |  | .64 |  |  |  |  |  |  |
| Me gusta jugar con gente online o en la misma habitación  *I like playing with people online or in the same room* |  | .64 |  |  |  |  |  |  |
| Así encajo en un grupo de gente que me gusta  *Thus, I fit in a group I like* |  | .63 | .31 |  |  |  |  |  |
| Otros jugadores me valoran en el juego  *Other players appreciate me in the game* |  | .49 |  |  |  |  |  |  |
| Así tengo cosas de las que hablar con mis amigos  *Thus I have things to talk about with my friends* |  | .45 |  |  |  |  |  |  |
| Tengo prestigio por mis éxitos en el juego  *I have prestige because of my successes in the game* |  | .42 |  |  |  |  |  |  |
| Gusto a los demás si juego  *The others like me if I play* |  | .40 |  |  |  |  |  |  |
| Todos mis amigos juegan  *All my friends play* |  | .40 |  |  |  |  |  |  |
| Me ayuda a olvidar problemas del día a día  *It help me forget my daily problems* |  |  | .70 |  |  |  |  |  |
| Olvido mis preocupaciones  *I forget my worries* |  |  | .69 |  |  |  |  |  |
| Me permite escapar del mundo real  *It allowes me to escape from the real world* |  |  | .66 |  |  |  |  |  |
| Me permite sentirme mejor cuando estoy frustrado  *Gaming allowes me to feel better when I am frustrated* |  |  | .44 |  |  |  |  |  |
| Me ayuda a mejorar mi estado de ánimo  *Gaming helps me to improve my mood* |  |  | .43 |  |  |  |  |  |
| Me ayuda a liberar mi energía negativa  *It helps to release negative energy* |  |  | .41 |  |  |  |  |  |
| Así no me siento excluido  *Thus I do not feel excluded* |  |  | .37 |  |  |  |  |  |
| Cuando estoy enfadado o disgustado con alguien, mediante el juego evito discutir con esa persona  *When I am angry or upset with someone, through gaming I avoid arguing with such person* |  |  | .34 |  |  |  |  |  |
| Disfruto de las peleas y luchas violentas en el juego  *I enjoy the violent fights in video game* |  |  |  | .88 |  |  |  |  |
| Me gusta la violencia en el juego, cuanto más mejor  *I like violence in video games, the more violent the better* |  |  |  | .86 |  |  |  |  |
| En el juego es divertido disparar a alguien en la cabeza  *Shooting someone in the head in a game is deeply satisfying* |  |  |  | .72 |  |  |  |  |
| Disfruto destrozando cosas en el juego  *I enjoy destroying thing in the game* |  |  |  | .68 |  |  |  |  |
| Matando en el juego me siento poderoso  *Killing in the game I feel powerful* |  |  |  | .59 |  |  | .34 |  |
| Me permite hacer cosas que no puedo hacer en la vida real  *It allowes me to do things I cannot do in the real life* |  |  |  | .44 |  |  |  |  |
| Me ayuda a canalizar mi agresividad  *It helps me channel my aggressivity* |  |  |  | .40 |  |  |  |  |
| Incrementan mis niveles de adrenalina  *Games increase my adrenalin levels* |  |  |  | .37 |  |  |  |  |
| Me siento inmerso en un mundo fantástico/ficticio  *I feel immersed in a fantastic/fictitious world* |  |  |  |  | .62 |  |  |  |
| Me gusta sentirme parte de una historia  *I like feeling myself part of a story* |  |  |  |  | .51 |  |  |  |
| Disfruto metiéndome en la piel de un personaje en cada juego  *I enjoy putting myself into a new character’s shoes in each game* |  |  |  |  | .47 |  |  |  |
| Me siento alguien importante en el juego  *I feel someone important in the game* |  |  |  |  | .43 |  |  |  |
| Me gusta explorar el mundo del juego para descubrir cosas nuevas  *I like to explore the world to find out new things* |  |  |  |  | .41 |  |  |  |
| Estimulan mis emociones  *Games stimulate my emotions* |  |  |  |  | .38 |  |  |  |
| Son excitantes y emocionantes  *Games are exciting* |  |  |  |  | .31 |  |  |  |
| Disfruto diseñando cosas en el juego  *I enjoy customizing things in the game* |  |  |  |  |  | .88 |  |  |
| Me gusta crear cosas en el juego, como casas u otras construcciones  *I like making things in video games, such as houses or other constructions* |  |  |  |  |  | .88 |  |  |
| Me gusta crear mi propio mundo en el juego  *I like to create my own world in games* |  |  |  |  |  | .85 |  |  |
| En el juego me gusta utilizar distintos elementos para crear nuevas cosas  *In the game I like to use different elements to create new things* |  |  |  |  |  | .76 |  |  |
| Me gusta diseñar o personalizar la apariencia de mis personajes  *I like designing or customizing the appearance of my characters* |  |  |  |  |  | .71 |  |  |
| Me agrada derrotar a otros jugadores  *I like to defeat other players* |  |  |  |  |  |  | .73 |  |
| Me gusta ganar  *I like to win* |  |  |  |  |  |  | .72 |  |
| Me gusta demostrar que soy mejor que otros jugadores  *I like to prove I am better than other players* |  |  |  |  |  |  | .71 |  |
| Me gusta provocar o picar a otros jugadores  *I like to provoke other players* |  |  |  |  |  |  | .52 |  |
| Me siento poderoso en el juego  *I feel myself powerful in the game* |  |  |  |  |  |  | .49 |  |
| Disfruto compitiendo con otros  *I enjoy competing with others* |  | .37 |  |  |  |  | .48 |  |
| Me suponen un reto mental  *Games imply a mental challenge* |  |  |  |  |  |  |  | .75 |
| Me hacen más inteligente  *Games make me smarter* |  |  |  |  |  |  |  | .69 |
| Me hacen pensar/calentarme la cabeza  *Games make me thing* |  |  |  |  |  |  |  | .59 |
| Agudizan mis sentidos  *Games sharpen my senses* |  |  |  |  |  |  |  | .56 |
| Me activan  *Games trigger me* |  |  |  |  |  |  |  | .53 |
| Mejoran mis habilidades  *Games improve my abilities* |  |  |  |  |  |  |  | .50 |

*Note*. Only factors loadings higher than .30 were presented.

Items with factor loading lower than .30 were not depicted: Me gustan las emociones intensas (*I like intense feelings*), Así los demás no se burlarán de mí ni se meten conmigo por no jugar (*Thus the others will not mock me because no* *gaming*), Me siento orgulloso de mi actuación en el juego (*I feel proud of my in-game performance*), Me ayuda a ser menos violento en la vida real (*Games help me to be less violent in the real life*).
