# Supplementary material for: The development and validation of the Videogaming Motives Questionnaire (VMQ)
Source: PLoS One. 2020 Oct 23;15(10):e0240726. doi: 10.1371/journal.pone.0240726 (PMC7584249; doi:10.1371/journal.pone.0240726)
Supplement: S2 Table — (DOCX) [file pone.0240726.s002.docx]

**S2 Table. Study 2: Descriptive data by gender, gender differences and correlation matrix**

|  | Males’ mean (SD) | Females’ mean (SD) | Sign. *t* | Cohen’s *d* | 1 | 2 | 3 | 4 | 5 | 6 | 7 | 8 | 9 |
| --- | --- | --- | --- | --- | --- | --- | --- | --- | --- | --- | --- | --- | --- |
| 1. Recreation | 10.43 (2.06) | 9.64 (2.15) | .001 | .38 | - |  |  |  |  |  |  |  |  |
| 2. Competition | 8.30 (2.59) | 5.31 (2.95) | .000 | 1.08 | .42** | - |  |  |  |  |  |  |  |
| 3. Cognitive development | 5.93 (3.26) | 4.50 (3.03) | .000 | .45 | .37** | .53** | - |  |  |  |  |  |  |
| 4. Coping | 7.10 (3.41) | 5.76 (3.64) | .000 | .38 | .44** | .48** | .53** | - |  |  |  |  |  |
| 5. Social interaction | 5.70 (3.29) | 3.23 (2.98) | .000 | .79 | .28** | .57** | .58** | .50** | - |  |  |  |  |
| 6. Violent reward | 6.54 (3.33) | 3.09 (3.70) | .000 | .98 | .27** | .49** | .30** | .37** | .41** | - |  |  |  |
| 7. Customization | 6.14 (3.66) | 6.60 (3.90) | .250 | .12 | .26** | .18** | .39** | .34** | .27** | .15** | - |  |  |
| 8. Fantasy | 6.24 (3.37) | 5.16 (3.54) | .004 | .31 | .46** | .36** | .57** | .57** | .44** | .34** | .55** | - |  |
| 9. Gaming hours | 19.49 (14.41) | 11.16 (8.71) | .000 | .70 | .23** | .32** | .24** | .34** | .37** | .28** | .19** | .26** | - |
| 10. Disordered gaming | 5.81 (5.52) | 2.87 (3.63) | .000 | .63 | .18** | .39** | .37** | .48** | .51** | .36** | .18** | .31** | .42** |

Note. Gender differences, Student t test.

Cohen’s *d* effect size: small, .20; medium, .50; large, .80.
**p*<.05*, **p*<.01
