# Supplementary material for: The development and validation of the Videogaming Motives Questionnaire (VMQ)
Source: PLoS One. 2020 Oct 23;15(10):e0240726. doi: 10.1371/journal.pone.0240726 (PMC7584249; doi:10.1371/journal.pone.0240726)
Supplement: S3 Table — (DOCX) [file pone.0240726.s003.docx]

**S3 Table. Study 3: Descriptive data by gender, gender differences and correlation matrix**

|  | Males’ mean (SD) | Females’ mean (SD) | Sign. *t* | Cohen’s *d* | 1 | 2 | 3 | 4 | 5 | 6 | 7 | 8 | 9 |
| --- | --- | --- | --- | --- | --- | --- | --- | --- | --- | --- | --- | --- | --- |
| 1. Recreation | 13.85 (1.77) | 12.57 (2.25) | .000 | .63 | - |  |  |  |  |  |  |  |  |
| 2. Competition | 11.21 (2.86) | 8.88 (3.17) | .000 | .77 | .39** | - |  |  |  |  |  |  |  |
| 3. Cognitive development | 10.28 (2.71) | 9.23 (3.16) | .010 | .36 | .39** | .24** | - |  |  |  |  |  |  |
| 4. Coping | 9.79 (2.91) | 8.78 (3.27) | .019 | .33 | .48** | .29** | .44** | - |  |  |  |  |  |
| 5. Social interaction | 8.34 (3.21) | 5.70 (2.92) | .000 | .86 | .38** | .41** | .34** | .38** | - |  |  |  |  |
| 6. Violent reward | 8.48 (3.40) | 5.18 (2.94) | .000 | 1.04 | .29** | .34** | .16* | .32** | .27** | - |  |  |  |
| 7. Customization | 9.53 (3.39) | 10.22 (4.03) | .174 | .19 | .36** | .12 | .27** | .35** | .17* | .13 | - |  |  |
| 8. Fantasy | 10.39 (3.34) | 9.10 (4.05) | .013 | .33 | .51** | .22** | .40** | .47** | .30** | .40** | .60** | - |  |
| 9. Gaming hours | 14.09 (13.89) | 7.09 (10.63) | .000 | .57 | .29** | .15* | .12* | .24** | .42** | .21** | .15* | .24** | - |
| 10. Disordered gaming | 38.39 (11.55) | 30.48 (9.27) | .000 | .76 | .29** | .28** | .21** | .39** | .41** | .43** | .10 | .34** | .43** |

Note. Gender differences, Student t test.

Cohen’s *d* effect size: small, .20; medium, .50; large, .80.
**p*<.05*, **p*<.01
