# Supplementary material for: The development and validation of the Videogaming Motives Questionnaire (VMQ)
Source: PLoS One. 2020 Oct 23;15(10):e0240726. doi: 10.1371/journal.pone.0240726 (PMC7584249; doi:10.1371/journal.pone.0240726)
Supplement: Attachment — Submitted filename: PONE-D-20-12358-rev.pdf [file pone.0240726.s004.pdf]

Ms #: PONE-D-20-12358

Title: The development and validation of the Videogaming Motives Questionnaire (VMQ)

Authors: López F, et al.

Summary:

This study examined the factor structure of a new instrument, the Videogaming Motives Questionnaire, in three samples of Spanish adolescents and young adults. Study 1 used exploratory factor analysis (EFA) to define empirical factors in young adults. The EFA revealed that an 8-factor model best explained the data. This pattern was replicated in Study 2 in a sample of adolescents. Finally, Study 3 used confirmatory factor analysis to demonstrate a selective mapping/loading of items to the 8 respective motive factors. Overall psychometric properties indicated clear simple structure and internal reliability of all factors. The sample size was sufficient to conclude the models are stable. Coping was identified as a key motive linked with gaming duration and with disordered gaming (DG), based on an author-compiled index of DG. Social interaction was also strongly linked with heavier gaming and DG. The latter result may reflect a preference for on-line socializing by people who are not entirely comfortable with direct interpersonal encounters. Violent motives also coincided with DG, while male gender coincided with stronger endorsement of all motives, aside from Customization, and DG, consistent with other addictive behaviors. The Discussion interpreted the findings for each of the key motives and proposed possible reasons for the pattern of effects.

Comments/Questions:

1. Ms p. 3, line 51

The term "gaming addiction" is used along with a number of others (disordered gaming, pathological video gaming, disordered gaming behavior) to describe the syndrome under investigation. For simplicity, it would be helpful if the authors select a single term to describe the syndrome and use that consistently throughout the manuscript. They might even mention, parenthetically, that they will be using this term to describe the target syndrome, the first time it is introduced.

2. Ms p. 7 - line 103

The term "IGD" is introduced but not defined. Perhaps Internet Gaming Disorder (IGD) is the term they wish to use as their standard description of the syndrome?

3. Table 1 provides a very clear summary of the findings to date.

4. Ms p. 8, lines 129-130:

"All these deficiencies impede..." It is not clear that these are "deficiencies" as opposed to different conceptual or explanatory frameworks or preferred terminology. Perhaps a more neutral description (e.g., "This variability across the different gaming scales impedes...") would work equally well?

5. In the Introduction, it would be helpful to specify the age of “adolescents” (12-17 years)? "young adults" (18-25 years)?

6. Page 8 - When describing the Participants, it would be helpful to report any incentive offered or alternative reason to participate in the study, or if none was offered, this could be stated as well. Same for studies 2 and 3.

6. Ms p. 9, line 151 "recruited from the LAN party" The term LAN should be defined for general readership

7. Ms p. 9, line 156 "Through an exhaustive scoping review of the literature"

It would be helpful to specify which databases were examined, the years of publication deemed eligible for inclusion, and a sample of the search terms used.

8. Ms p 9, line 172

What was the rationale for using oblique as opposed to orthogonal rotation to achieve simple structure in the two EFAs?

9. Ms p 11, lines 220-2

Weekly number of gaming hours was obtained by adding online and offline daily hours and multiplying the days of these periods of the week by the total number of hours. Global retrospective estimates of time engaged in activities like drinking or gambling have been found to be less accurate than values based on a Timeline Followback (see Sobell and Sobell 1992) procedure. Since Timeline Followback was not mentioned here, the limitations of the current reporting procedure should be acknowledged in the Discussion

10. Ms p 12, line 246

Hierarchical multiple regressions were used to examine the relationship between motives, gaming intensity and problems, while controlling for age and gender. What was the method of entry of predictors in each stage, and briefly what was the rationale for this?

11. Ms p. 18

Study 3 refers to the Emerging Adulthood sample. This is the only time this terminology is used. Were the age inclusion criteria different than for study 2? If not, perhaps it is best to stick with 'Young Adult' as the description?

12. Ms p. 21

In the Discussion, before launching into the specific motives that emerged, it would be good to state that an 8-factor model appeared to best explain the data in all 3 samples, and to report the proportion of variance explained by these factors in each case. The first paragraph under Conclusion, p 26 addresses many of these points and could be moved up to the beginning of this section.

Some comment on the inter-correlation vs. orthogonality of factors might also be made and what this overall pattern of scores means for VMQ as assessment tool, generally. The consistent elevation in motive endorsement by males, aside from customization, may also merit some comment.

13. As the authors note, Coping emerged as a reliable and somewhat selective predictor of disordered gaming (as compared with Social Interaction, for example). Although this makes intuitive sense, I think this linkage, which, again as noted, is seen with other addictive behaviors, might be considered a bit more with respect to addiction pathology.

For example, what is the difference between wanting to engage in a behavior and needing to do so?

How do these different global motivations correspond to the concept of "dependence" within the addiction framework?

And what would be the expected role of environmental factors like "stress" as a conditioned (i.e., involuntary) cue for gaming in people with these different motivational profiles?

<u>Minor Points:</u>

1. Ms p. 4, line 71 "based on well-stablished theories" Typo: well-[e]stablished

2. Ms p. 7, line 119 "the described motives suffer from [?] of ‘jingle-jangle’ fallacies," Missing words [a form]

3. Table 2, Cognitive Development factor: Item 3 (Games make me thing). Typo - Games make me thin[k]

4. Ms p. 19, line 320 "we used the S−Bχ2/df to overcome such problem [74]." Syntax "to overcome [this] problem"
