# Supplementary material for: The development and validation of the Videogaming Motives Questionnaire (VMQ)
Source: PLoS One. 2020 Oct 23;15(10):e0240726. doi: 10.1371/journal.pone.0240726 (PMC7584249; doi:10.1371/journal.pone.0240726)
Supplement: Attachment — Submitted filename: Response To Reviewers.docx [file pone.0240726.s005.docx]

**Journal Requirements:**

When submitting your revision, we need you to address these additional requirements.

1. Please ensure that your manuscript meets PLOS ONE's style requirements, including those for file naming. The PLOS ONE style templates can be found at

<https://journals.plos.org/plosone/s/file?id=wjVg/PLOSOne_formatting_sample_main_body.pdf> and

<https://journals.plos.org/plosone/s/file?id=ba62/PLOSOne_formatting_sample_title_authors_affiliations.pdf>

Response: Done

2. Please include additional information about your scoping review in your methods section, including search strategy, inclusion and exclusion criteria, to enable reproducibility and replicability.

Response: Dear editor,

We regret to inform you that there was a translation misunderstanding (an example of “lost in translation”) with regard to the term “scoping review”. We conducted an extensive review of motivational scale studies, selecting most of the relevant questionnaires used in research, but it was not a scoping review. Later, when the English language revision of the paper was carried out, “extensive review” was changed to “scoping review”. This was unfortunate as these concepts are different. We would like to apologize again. Accordingly, we have changed the term “scoping review” for the original “extensive review”. In any case, we have described in more detail which databases were examined, the years of publication eligible for inclusion, and a sample of the search terms used, as suggested (see **p. 9, lines 160-165**)

3. Please proofread your manuscript for typos (for instance item 3 of table 2).

Response: Done

4. Your ethics statement must appear in the Methods section of your manuscript. If your ethics statement is written in any section besides the Methods, please move it to the Methods section and delete it from any other section. Please also ensure that your ethics statement is included in your manuscript, as the ethics section of your online submission will not be published alongside your manuscript.

Response: Done

**Response to reviewer:**

We really appreciate the reviewer’s contribution.

1. Ms p. 3, line 51

The term "gaming addiction" is used along with a number of others (disordered gaming, pathological video gaming, disordered gaming behavior) to describe the syndrome under investigation. For simplicity, it would be helpful if the authors select a single term to describe the syndrome and use that consistently throughout the manuscript. They might even mention, parenthetically, that they will be using this term to describe the target syndrome, the first time it is introduced.

Response: **p. 3, line 53-54**

“These inclusions in the DSM-5 and ICD-11 assume that disordered gaming, (for simplicity, this term will be used throughout the manuscript to describe the syndrome)… “

2. Ms p. 7 - line 103

The term "IGD" is introduced but not defined. Perhaps Internet Gaming Disorder (IGD) is the term they wish to use as their standard description of the syndrome?

Response: **p. 3, line 47-49**

… defined as a “*persistent and recurrent use of the Internet to engage in games, often with other players, leading to clinically significant impairment or distress ...*”

3. Table 1 provides a very clear summary of the findings to date.

4. Ms p. 8, lines 129-130:

"All these deficiencies impede..." It is not clear that these are "deficiencies" as opposed to different conceptual or explanatory frameworks or preferred terminology. Perhaps a more neutral description (e.g., "This variability across the different gaming scales impedes...") would work equally well?

Response: **p. 8, lines 132-133**

“This variability across the different gaming scales impedes…”

5. In the Introduction, it would be helpful to specify the age of “adolescents” (12-17 years)? "young adults" (18-25 years)?

Response: **p. 3, line 43**

“… adolescents (12-17 years) and young adults (18-24 year)”

6. Page 8 - When describing the Participants, it would be helpful to report any incentive offered or alternative reason to participate in the study, or if none was offered, this could be stated as well. Same for studies 2 and 3.

Study 1

Response: **p. 9, line 156**

“Participants voluntarily completed an online survey without any compensation”

Study 2

Response: **p. 11, line 210**

“… and received school supplies as an incentive”

Study 3

Response: **p. 19, line 323**

“The survey was selflessly completed online”

6. Ms p. 9, line 151 "recruited from the LAN party" The term LAN should be defined for general readership

Response: **pp. 8-9, lines 153-154**

“.. an event at the Jaume I University in which gamers gathered to play multiplayer video games, ..”

7. Ms p. 9, line 156 "Through an exhaustive scoping review of the literature"

It would be helpful to specify which databases were examined, the years of publication deemed eligible for inclusion, and a sample of the search terms used.

Response: **p. 9, lines 160-165**

Through an extensive review of the literature concerning gaming motivation scales, 25 relevant gaming motive scales were selected. For the review, it was conducted a search in the databases PsycINFO and Google Scholar from 2000 to present, combining the key words motives or motivation, video game or gaming, scale or questionnaire. Furthermore, citations from selected studies of our database were used to continue the review process until no other relevant study was found.

8. Ms p 9, line 172

What was the rationale for using oblique as opposed to orthogonal rotation to achieve simple structure in the two EFAs?

Response:

Oblimin rotation (which does not assume prior relations between factors and allows axes to freely rotate) is somewhat more exploratory than orthogonal rotation (which assumes independence between factors and forces axes to be orthogonal), and also allows to estimate the intercorrelations between factors. Based on previous psychometric studies on motives for gaming and other addictive behaviors, we expected high intercorrelations between factors. Accordingly, oblimin rotation was used in the pilot study. The pilot study confirmed that factors were highly intercorrelated, so the oblimin rotation was also used in the EFA of the second study, and the correlations between factors were specified in the CFA of the third study.

Thus, we have added in Statistical Analysis section of study 2:

**p. 13, lines 255-257** “As the pilot study showed that motives were intercorrelated, an EFA with principal axis factoring and oblique rotation (oblimin) was performed for testing the VMQ with original eight-factor model using IBM SPSS Statistics V21.0.”

9. Ms p 11, lines 220-2

Weekly number of gaming hours was obtained by adding online and offline daily hours and multiplying the days of these periods of the week by the total number of hours. Global retrospective estimates of time engaged in activities like drinking or gambling have been found to be less accurate than values based on a Timeline Followback (see Sobell and Sobell 1992) procedure. Since Timeline Followback was not mentioned here, the limitations of the current reporting procedure should be acknowledged in the Response: Discussion

**p. 29, lines 562-564**

**“**Fourth, the time engaged in gaming was obtained by self-report retrospective estimates, which have been found to be less accurate than other procedures such as Timeline Followback [82]”.

10. Ms p 12, line 246

Hierarchical multiple regressions were used to examine the relationship between motives, gaming intensity and problems, while controlling for age and gender. What was the method of entry of predictors in each stage, and briefly what was the

rationale for this?

Response: **p. 13, lines 263-267**

“Previous research has shown that gender and age were relevant variables in video game–related behaviors, such as disordered use or video game genre preferences (e.g., [9,32,57]). Therefore, hierarchical multiple regressions were controlled for gender and age by introducing them in step 1, whereas motives were entered in step 2”.

11. Ms p. 18

Study 3 refers to the Emerging Adulthood sample. This is the only time this terminology is used. Were the age inclusion criteria different than for study 2? If not, perhaps it is best to stick with 'Young Adult' as the description?

Response: **p. 19, line 318**

“Study 3. Young adult sample study”

12. Ms p. 21

In the Discussion, before launching into the specific motives that emerged, it would be good to state that an 8-factor model appeared to best explain the data in all 3 samples, and to report the proportion of variance explained by these factors in each case.

AND

The first paragraph under Conclusion, p 26 addresses many of these points and could be moved up to the beginning of this section.

AND

Some comment on the inter-correlation vs. orthogonality of factors might also be made and what this overall pattern of scores means for VMQ as assessment tool, generally.

AND

The consistent elevation in motive endorsement by males, aside from customization, may also merit some comment.

Response: **p. 22., lines 402-405**

“As a result, the VMQ consisted of an 8-intercorrelated-factor model that appeared to best explain the data. It showed good internal consistency and temporal stability reliability indices, and also presented evidence of validity when predicting video game frequency of use and disordered gaming.”

And **the information of % of variance is now presented at the Result sections of each study (Factor Structure)**

**Initial testing:** 78.7%; **Adolescent sample study:** 78.5% & **Young adulthood sample**: 81.9%

**p. 28, lines 546-556**

“Regarding gender, adolescent and young adult males presented higher scores than females in nearly all scales apart from customization, especially in competitive, violent reward and social interaction motives. These higher motivational levels in male gender for almost all motives has been systematically found in previous studies (e.g., [32-36, 57]). Along these lines, it has been reported that adolescent males prefer competitive and violent game genres, such as action-*shooters*, sport, fight or strategy games; whereas girls prefer game genres such as brain and skill games, and social simulation games, more characterized by customization aspects [9]. These gender-based differences on motives for gaming may help to explain why males, who prefer games featuring more time-consuming and engagement characteristics, presented higher prevalence of regular and disordered use of video games than females [7].”

13. As the authors note, Coping emerged as a reliable and somewhat selective predictor of disordered gaming (as compared with Social Interaction, for example). Although this makes intuitive sense, I think this linkage, which, again as noted, is seen with other addictive behaviors, might be considered a bit more with respect to addiction pathology.

For example, what is the difference between wanting to engage in a behavior and needing to do so?

How do these different global motivations correspond to the concept of "dependence" within the addiction framework? And what would be the expected role of environmental factors like "stress" as a conditioned (i.e., involuntary) cue for gaming in people with these different motivational profiles?

Response: We have tried to reflect all these very insightful considerations in the following paragraph:

**p. 23, lines 426-434**

“In the present study, coping was the strongest and somewhat selective predictor of disordered gaming scores among both adults and adolescents. This result is in accordance with previous studies in which escape/coping motives presented the highest association with disordered gaming [48-50,52,53,59,77] and with problematic substance use and gambling [22-27]. This motive appears to be especially relevant in the presence of psychological problems and environmental stressors, such as depression and anxiety symptoms [50, 54-56] or stressful life events [78]. Accordingly, motivation for escape or relief from negative mood constitutes an important criterion for the diagnoses of IGD [5] and other addictions in the DSM-5 [3]

Minor Points:

Done
