# Supplementary material for: The development and validation of the Videogaming Motives Questionnaire (VMQ)
Source: PLoS One. 2020 Oct 23;15(10):e0240726. doi: 10.1371/journal.pone.0240726 (PMC7584249; doi:10.1371/journal.pone.0240726)
Supplement: Attachment — Submitted filename: Response To Reviewers.docx [file pone.0240726.s006.docx]

Additional Editor Comments:

Dear Authors:

I appreciate the strength of the paper and I believe that the changes made in response to the reviewer’s comments contributed to improve it. However, I have a minor comment about the scales you used to measure disordered gaming. You employed two different scales in Study 2 and Study 3, so I believe that this point deserves some explanation. Could you motivate your choice? Did this choice have an impact on your results? Additionally, was the Gentile’s scale validated in Spanish (as the IGD-20 )? If not, how do you translate it?

**Response**:

Dear Editor:

Thanks for your comments.

We employed different scales to measure disordered gaming in each study because of the sample’s age. The scale used in the adolescent sample of Study 2 was originally developed for the assessment of gaming disorders in youths aged from 8 to 18 years (see Gentile, 2009), and validated in Spain with an adolescent sample (the mean age of the validation sample in Spain was 14,29 years old; see López-Fernández et al., 2020). In the sample of young adults of Study 3, we considered a better option to use the IGD-20, originally developed in a sample of young adults (Pontes et al., 2014) and validated two years later in Spain (Fuster et al., 2016) (mean age of the original sample of 26.5 years old, and 26.14 years old in in the Spanish sample).

Although these scales somewhat different, both were based on similar DSM criteria, so we consider that this difference in the assessment of gaming disorder would have a limited impact on the results. This idea has been reinforced by the fact that disordered gaming assessed with the different scales have converged in finding that the most important motives for disordered gaming have been coping, social interaction and violent reward (in the same order). In any case, we have added a new limitation:

“Five, two different scales was employed for assessing disordered gaming in adolescents and young adults. Although we have found that main motives related to disordered gaming were the same in both samples, some of the differences found between adolescents and young adults could be attributed to the different scales used.”

Last, the Gentile’s scale usen in study 2 was validated in Spanish adolescents in a previous study (López-Fernández et al., 2020). Extracted from López-Fernández et al., 2020:

“The original 11 items was back-translated… For the study, the participants indicated their frequency of video game-related problems on a 4-point Likert scale (from 0 = “never or almost never” to 3 “almost always or always”) during the last 12 months. According to the parallel analysis run using Monte Carlo PCA (Watkins, 2006), a one-factor structure was obtained with the EFA, where all items presented adequate factor loadings ranging from .49 to .81. Cronbach’s alpha in this sample was .88”.

Thus, now we have included this fact in the text as follows:

**p. 13, lines 248-249**

“A Spanish adaptation [9] of a measure especially developed to assess disordered gaming in adolescents [70] was employed”.
